# A two-minute exercise snack may be sufficient to enhance energy metabolism and fat oxidation in sedentary male college students

**DOI:** 10.3389/fphys.2026.1929244

**Published:** 2026-09-11

**Authors:** Zhihao Gao, Yanbai Han, Yongbo Wang, Zhuoyue Cheng, Zhiyu Xiang, Yiming Han

**Affiliations:** College of Physical Education and Health, Guangxi Normal University, Guilin, China

**Keywords:** energy expenditure, exercise snack, fat oxidation, sedentary behavior, substrate metabolism

## Abstract

**Objective:**

Short-duration exercise snacks are considered a time-efficient strategy for interrupting sedentary behavior, but the metabolic effects of different exercise durations remain unclear. This study compared energy expenditure and substrate oxidation among the 1-, 2-, and 3-min exercise snack protocols during exercise and a 30-min recovery period under a standardized target workload.

**Methods:**

Twenty sedentary male college students completed three exercise snack protocols in a randomized crossover design with a 7-day washout interval between trials. All protocols were performed on a cycle ergometer using a standardized incremental loading scheme (30 W every 10 s to 180 W). The 1-min protocol ended upon reaching 180 W, whereas the 2-min and 3-min protocols maintained this workload until exercise completion. Energy expenditure, fat oxidation, glucose oxidation, and associated physiological indices were continuously monitored during exercise and the 30-min recovery period.

**Results:**

All three exercise snack protocols elicited significant acute metabolic responses. During exercise, total fat oxidation was greater in the 2-min than in the 1-min protocol (0.71 ± 0.22 vs. 0.37 ± 0.12 g, p < 0.001), and was not significantly different from that observed in the 3-min protocol (0.71 ± 0.22 vs. 0.78 ± 0.34 g, p = 0.179). During recovery, energy expenditure rate declined in all protocols but followed different trajectories. In the 1-min protocol, it returned to resting level by 25 min (p = 0.095), whereas it remained above resting throughout the 30-min recovery period in the 2-min (1.87 ± 0.21 kcal/min) and 3-min protocols (2.07 ± 0.32 kcal/min; both p < 0.001). Fat oxidation rate increased whereas glucose oxidation rate decreased during recovery. At 30 min of recovery, fat oxidation rate was not significantly different between the 2-min and 3-min protocols (0.15 ± 0.02 vs. 0.17 ± 0.04 g/min, p = 0.068).

**Conclusion:**

A 2-min exercise snack may be sufficient to increase energy expenditure and fat oxidation while eliciting sustained post-exercise metabolic activation. These findings provide evidence to support the potential application of exercise snacks as a time-efficient exercise strategy for sedentary adults.

## 1 Introduction

With changes in modern lifestyles, daily sedentary time has increased markedly, and sedentary behavior has become a major global public health concern. Prolonged sedentary behavior and physical inactivity are closely associated with an increased risk of multiple chronic diseases, including cardiovascular disease, metabolic dysfunction, excess body weight, and poor mental health (Owen et al., 2020; Blodgett et al., 2024). Epidemiological evidence indicates that regular physical activity reduces the risk of chronic diseases and improves cardiometabolic health (Kazemi et al., 2024). Nevertheless, physical inactivity remains highly prevalent worldwide. The World Health Organization Global Action Plan on Physical Activity 2018–2030 aims to reduce the global prevalence of physical inactivity by 15% by 2030 relative to 2010 levels (World Health Organization, 2018; Bull et al., 2020). Accordingly, developing exercise interventions that are time-efficient, easy to implement, and fit seamlessly into daily routines is of considerable practical importance for improving metabolic health in sedentary populations.

Compared with traditional continuous exercise, short-duration exercise snacks are characterized by their brief duration, flexibility, and high adherence, making them a time-efficient strategy for interrupting prolonged sedentary behavior. In the present study, short-duration exercise snacks were operationally defined as acute exercise stimuli lasting ≤ 3 min that reached a predetermined target workload at submaximal intensity under a standardized incremental loading protocol. Previous exercise snack studies have employed a wide range of exercise modalities and intensities, ranging from light-intensity walking to vigorous stair climbing, which limits direct comparisons among studies and highlights the importance of standardized and reproducible protocols for clarifying the metabolic effects of exercise snacks. Compared with low-intensity activity, exercise performed at submaximal intensity elicits greater cardiopulmonary and metabolic responses within a short period while maintaining safety and feasibility, and has therefore been widely used in exercise physiology research and clinical exercise testing (Hoehn et al., 2015; Noonan and Dean, 2000). Previous studies have shown that submaximal-intensity exercise increases energy expenditure (EE) and promotes carbohydrate and fat metabolism, thereby improving cardiorespiratory fitness and metabolic health (Garber et al., 2011). Furthermore, incremental loading exercise provides excellent standardization and reproducibility by delivering a uniform exercise stimulus through a controlled loading progression, thereby providing a reliable experimental basis for comparing metabolic responses to exercise snacks of different durations (Iannetta et al., 2019).

Previous studies have examined brief activity bouts lasting approximately 1–3 min to interrupt prolonged sitting at intervals of about 30 min and have reported favorable cardiometabolic responses, particularly in relation to glycemic control (Smith et al., 2021; Yin et al., 2024c). These durations therefore align with the brief and practically feasible nature of exercise snack interventions. However, previous research has primarily focused on the frequency of sedentary interruptions and glycemic outcomes, whereas direct comparisons of different exercise snack durations in terms of EE and substrate oxidation remain scarce. Accordingly, the present study selected exercise durations of 1, 2, and 3 min as progressively longer conditions to examine whether extending exercise duration produces additional metabolic responses, to explore the minimum duration capable of eliciting meaningful acute and post-exercise metabolic responses, and to characterize the duration-response relationship during exercise and subsequent recovery under the same standardized loading protocol. Furthermore, no consensus has been reached regarding key exercise prescription parameters, including exercise modality, exercise intensity, and loading progression, which has hindered the standardization and wider implementation of exercise snack interventions (Cho et al., 2020; Benatti and Ried-Larsen, 2015).

The post-exercise recovery period is a critical phase for evaluating acute metabolic responses to exercise. Following exercise cessation, oxygen uptake (VO_2_) and EE remain elevated above resting levels for a period of time, a phenomenon commonly referred to as excess post-exercise oxygen consumption (EPOC). The magnitude and duration of EPOC are influenced by exercise intensity, exercise modality, and recovery conditions (Panissa et al., 2021; Sindorf et al., 2021). During recovery, dynamic changes in EE, fat oxidation, and glucose oxidation reflect the degree of post-exercise metabolic activation and shifts in substrate utilization (Jiang et al., 2024; Faleiro et al., 2025). Therefore, evaluating EE during exercise alone may be insufficient to fully characterize the metabolic effects of exercise snacks. A systematic assessment of EE and substrate oxidation during both exercise and recovery may further clarify whether exercise snacks can induce sustained metabolic activation despite their very short duration. However, evidence regarding the effects of exercise duration on post-exercise substrate oxidation remains limited, particularly with respect to direct comparisons of the recovery trajectories of fat oxidation and glucose oxidation following exercise snacks of different durations.

Accordingly, the present study employed a standardized incremental loading protocol on a cycle ergometer to compare the acute metabolic effects of 1-, 2-, and 3-min exercise snacks under a standardized target workload (180 W). Particular emphasis was placed on comparing EE, fat oxidation, and glucose oxidation among the 1-, 2-, and 3-min exercise snack protocols during exercise and throughout a 30-min recovery period. The aim of this study was to characterize differences among the exercise-duration protocols in post-exercise metabolic activation and substrate oxidation, thereby providing experimental evidence for optimizing time-efficient exercise interventions for sedentary adults. We hypothesized that, under a standardized target workload, exercise snacks of different durations would all elicit acute metabolic responses, with longer exercise durations producing greater EE and substrate oxidation during the recovery period.

## 2 Subjects and methods

A randomized crossover design was adopted in this study. All six possible sequences of the 1-, 2-, and 3-min exercise snack conditions were predefined, and each participant randomly drew one sequence before the formal experiment to determine the order of the three trials. A pilot study was conducted to determine an appropriate loading progression and target workload for the formal experiment. Loading increments of 10, 20, and 30 W every 10 s were evaluated. The 10- and 20-W incremental protocols elicited relatively limited metabolic responses within the brief exercise period. In contrast, the 30-W-per-10-s protocol progressing to 180 W produced a more discernible metabolic response while remaining feasible for completion during the pilot trials. Therefore, the 30-W-per-10-s incremental protocol with a target workload of 180 W was adopted for the formal experiment, providing a standardized loading procedure for direct comparisons of metabolic responses among the 1-, 2-, and 3-min exercise conditions.

The formal experiment included 20 sedentary male college students. After baseline assessments of height, body mass, resting heart rate (HR), fat mass, lean body mass, and body fat percentage, participants completed three independent exercise protocols on a cycle ergometer while wearing a portable metabolic system (Cosmed K5; Cosmed, Rome, Italy). A 7-day washout period was scheduled between consecutive trials to minimize potential carryover effects from the preceding exercise session (Wang Y. et al. (2024)). The three protocols consisted of 1-, 2-, and 3-min exercise snacks, respectively. All protocols employed an incremental loading rate of 30 W every 10 s until reaching a standardized target workload of 180 W. In the 2- and 3-min protocols, the target workload was maintained until the end of exercise. Following each exercise protocol, participants remained seated for a 30-min recovery period. Throughout the experiment, the Cosmed K5 portable metabolic system (Cosmed, Rome, Italy) continuously measured oxygen uptake (VO_2_) and carbon dioxide production (VCO_2_). Based on continuously collected VO_2_ and VCO_2_ data, indirect calorimetry was used to calculate EE, fat oxidation, and glucose oxidation during both exercise and recovery. In addition, changes in energy expenditure rate (EER), fat oxidation rate (FOR), and glucose oxidation rate (GOR) during the recovery period were further analyzed.

### 2.1 Participants

An *a priori* sample size calculation was performed using G*Power software (version 3.1.9.7) for a repeated-measures analysis of variance (ANOVA) across the three exercise protocols. Assuming an effect size of f = 0.25, a significance level of α = 0.05, and a statistical power of 0.80, the estimated minimum sample size required to detect a medium effect among the exercise protocols was 15 participants. Ultimately, 20 physically inactive and sedentary male college students were recruited, exceeding the minimum sample size requirement and providing sufficient statistical power for the present study. Baseline participant characteristics were as follows: body mass, 78.24 ± 9.26 kg; height, 1.74 ± 0.05 m; body mass index (BMI), 25.62 ± 3.61 kg/m²; body fat percentage, 24.00 ± 6.00%; fat mass, 19.00 ± 6.31 kg; and lean body mass, 59.24 ± 4.77 kg.

Participants were recruited through public advertisement. The inclusion criteria were as follows: (1) male college students aged 18–26 years; (2) a predominantly sedentary daily routine, determined during recruitment screening based on self-reported habitual behavior characterized by prolonged periods of sitting, reclining, or lying during study and leisure activities, consistent with the definition of sedentary behavior as an EE of ≤1.5 metabolic equivalents (METs) in these postures (Tremblay et al., 2017); (3) no regular exercise habit within the previous 3 months; (4) no participation in competitive sports or interval training; and (5) no history of smoking. The exclusion criteria included the presence of chronic diseases or other health conditions that could be induced or aggravated by exercise, as well as a background in sport-related disciplines or previous systematic exercise training.

All participants were instructed to refrain from vigorous exercise, sleep deprivation, smoking, alcohol consumption, caffeinated beverages, and the use of medications that could affect metabolism for 48 h before each trial. All assessments were conducted at least 2 h after a meal to minimize the potential influence of postprandial metabolic responses on the experimental outcomes. Before testing, all participants received a detailed explanation of the study objectives, experimental procedures, and potential risks, and provided written informed consent prior to participation.

### 2.2 Experimental instruments and equipment

The primary equipment used in this study included an Ergoselect 100 cycle ergometer (Ergoline, Germany), which was used to deliver the standardized incremental loading exercise protocols; a Cosmed K5 portable metabolic system (Cosmed, Rome, Italy), which continuously measured respiratory gas exchange, EE, and substrate oxidation during exercise and recovery; an InBody 770 body composition analyzer (InBody, Republic of Korea), which was used to assess body composition, including fat mass, lean body mass, and body fat percentage; and a calibrated electronic stadiometer-scale and HR monitor, which were used to measure baseline anthropometric characteristics and resting physiological parameters.

Although no independent repeatability assessment of the Cosmed K5 was conducted in the present study, its validity and reliability have been well established in previous studies of exercise metabolism (Winkert et al., 2020; DeBlois et al., 2021; Perez-Suarez et al., 2018). To minimize systematic measurement error, standardized quality control procedures were implemented throughout the study. Before each testing session, gas, volume, and ambient condition calibrations were performed in accordance with the manufacturer’s instructions, and instrument accuracy was regularly verified using a standard calibration gas mixture. The cycle ergometer was calibrated before each trial to ensure stable and accurate incremental workload delivery. In addition, all testing procedures were conducted by the same trained investigator under standardized environmental conditions to maximize the reliability and comparability of the data across trials.

### 2.3 Experimental control

Throughout the experiment, the laboratory temperature was maintained at 24–26 °C, with the relative humidity controlled between 40% and 60%. All participants were provided with the same pre-exercise lunch consisting of rice, beef, vegetables, and yogurt, and the meal composition was kept consistent across experimental visits. Exercise testing was conducted 2 h after lunch, and all trials commenced at 14:30 to minimize the potential influence of postprandial metabolic responses and circadian variation on metabolic outcomes. Before the first trial, all participants received standardized instruction on the operation of the cycle ergometer and the experimental procedures. The study was approved by the Human Research Ethics Committee of Guangxi Normal University (Approval No. 20250702002). All procedures were conducted in accordance with the relevant institutional requirements and local legislation, and written informed consent was obtained from all participants before participation.

### 2.4 Preliminary measurement

Before the experiment, baseline anthropometric measurements were obtained, including height, body mass, resting HR, fat mass, lean body mass, and body fat percentage. Body mass index (BMI) was calculated as body mass (kg) divided by the square of height (m²) and expressed as kg/m². Fat mass, body fat percentage, and lean body mass were assessed using an InBody 770 body composition analyzer. Resting HR was measured using a HR monitor after participants had remained seated quietly for at least 2 h after lunch. Resting HR served as an individual physiological baseline for evaluating HR responses during the exercise protocols, thereby providing a more accurate assessment of the intervention effects (Feng et al., 2024). Predicted maximal HR was estimated using the equation 208 − 0.7 × age and was used to assist in evaluating exercise intensity and recovery from fatigue (Tanaka et al., 2001). To ensure both an appropriate exercise stimulus and participant safety, the target exercise intensity was set at the submaximal intensity. HR and participants’ physiological responses were continuously monitored throughout the experiment, and testing was terminated immediately if any adverse symptoms or abnormal physiological responses occurred.

### 2.5 Experimental procedures

**Figure 1:**
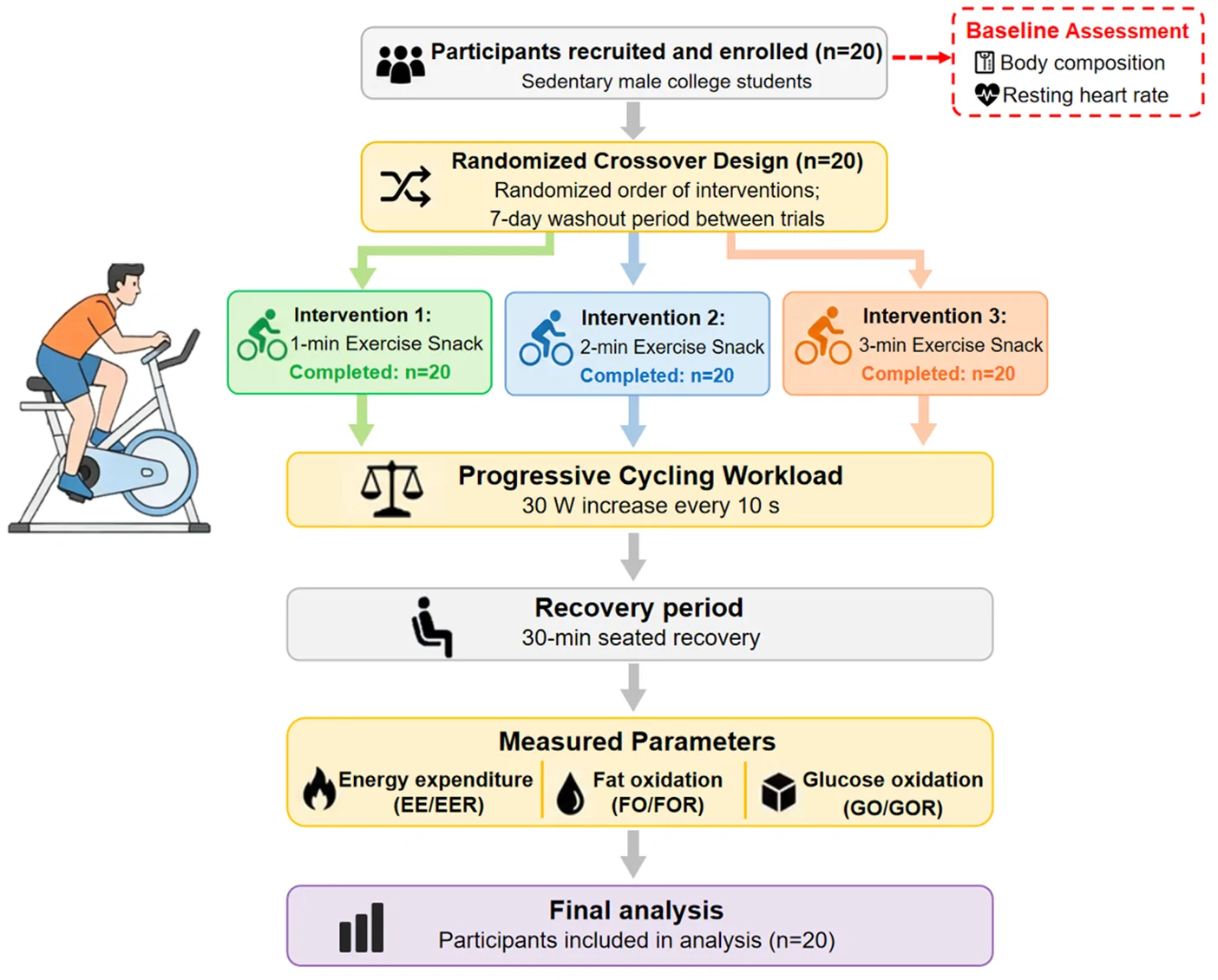
Experimental design and study procedure (n = 20). All 20 participants completed all three intervention periods and were included in the final analysis.

On each testing day, participants arrived at the laboratory at 14:20 and first completed a 10-min seated rest. During this period, the investigators provided standardized instructions and demonstrations regarding: (1) the operation of the cycle ergometer and precautions during exercise; (2) the correct fitting and use of the Cosmed K5 system; and (3) the placement of the HR monitor for continuous HR monitoring throughout the exercise protocol; and (4) the Borg Rating of Perceived Exertion (RPE) scale, including the meaning of the rating levels and how to report perceived exertion during exercise and recovery. Participants were then fitted with the HR monitor and the Cosmed K5 system and remained seated for an additional 5 min to collect resting metabolic data as the baseline measurement. Before the formal exercise protocol, participants performed a standardized 1-min warm-up on the cycle ergometer at a cadence of 60 r/min without external resistance, followed immediately by the formal exercise test. After completion of the exercise protocol, participants continued wearing the respiratory mask, and respiratory gas exchange was continuously monitored throughout the 30-min seated recovery period (**Figure 1**).

## 3 Measurements

### 3.1 Physiological indices measurement

During exercise and recovery, oxygen uptake (VO_2_, L/min) and carbon dioxide production (VCO_2_, L/min) were continuously measured using the Cosmed K5 portable metabolic system. The following equations were applied (Frayn, 1983; Trombold et al., 2014):

1. Glucose oxidation rate (g/min) = 4.585 × VCO_2_ (L/min) − 3.2256 × VO_2_ (L/min)
2. Glucose oxidation amount (g) = Glucose oxidation rate × Time (min)
3. Fat oxidation rate (g/min) = 1.695 × VO_2_ (L/min) − 1.701 × VCO_2_ (L/min)
4. Fat oxidation amount (g) = Fat oxidation rate × Time (min)
5. Energy expenditure rate (kcal/min) = 3.716 × VO_2_ (L/min) + 1.332 × VCO_2_ (L/min)
6. Energy expenditure (kcal) = [3.716 × VO_2_ (L/min) + 1.332 × VCO_2_ (L/min)] × Time (min)

Because substrate oxidation was estimated from VO_2_ and VCO_2_ using indirect calorimetry, transient non-steady-state gas-exchange responses during exercise or early recovery could yield physiologically implausible negative estimates of fat or glucose oxidation, a recognized limitation of this approach (Jeukendrup and Wallis, 2005). Therefore, negative calculated oxidation values were set to zero before subsequent analyses rather than being interpreted as actual negative substrate oxidation. The same processing procedure was applied consistently across all participants and experimental conditions. In addition, HR was recorded at 1-min intervals throughout the experimental session. RPE was assessed using the Borg scale during exercise and at 10, 20, and 30 min post-exercise to evaluate perceived exertion and recovery (Borg, 1982).

### 3.2 Statistical analysis

All statistical analyses were performed using SPSS 26.0 software (IBM, Chicago, IL, USA). Data are presented as mean ± standard deviation (mean ± SD). The normality of all variables was assessed using the Shapiro–Wilk test. Differences in outcome variables among the three exercise protocols during the exercise period and each of the 10-, 20-, and 30-min recovery periods were analyzed using one-way repeated-measures analysis of variance (ANOVA). To assess pre-trial metabolic stability across experimental periods, resting VO_2_ measured before the first, second, and third experimental visits was compared using one-way repeated-measures ANOVA. For variables assessed at multiple time points during recovery, the overall effects of exercise protocol and recovery time, as well as their interaction, were analyzed using two-way repeated-measures ANOVA, with exercise protocol and recovery time treated as within-subject factors. Mauchly’s test of sphericity was performed for all repeated-measures ANOVAs. When the assumption of sphericity was violated (p < 0.05), the Greenhouse–Geisser correction was applied. When a significant main effect or interaction was detected (p < 0.05), pairwise comparisons were performed using the Bonferroni adjustment. Effect sizes were expressed as partial eta squared (partial η²) and interpreted according to Cohen’s guidelines (1988): partial η² ≥ 0.14, large effect; 0.06 ≤ partial η² < 0.14, medium effect; and partial η² < 0.06, small effect (Cohen, 1988). Statistical significance was set at α = 0.05.

## 4 Results

EE and substrate metabolism are important indicators for evaluating exercise-induced metabolic changes during exercise. In the present study, fat oxidation and glucose oxidation were selected as the primary indicators of substrate metabolism to compare the effects of short-duration exercise snack protocols with different durations on fat oxidation and glucose oxidation rates. EER was used as the primary outcome to systematically evaluate the characteristics of energy metabolism under different exercise protocols. By monitoring EE and substrate metabolism during exercise, throughout the recovery period, and across the entire recovery process under the three exercise protocols, differences in EE and substrate utilization among the different exercise protocols were analyzed. The results showed that all exercise protocols elicited varying degrees of metabolic responses during both the exercise and recovery periods.

Resting VO_2_ did not differ significantly across the three experimental visits (Visit 1: 410.31 ± 51.76 mL/min; Visit 2: 421.84 ± 51.02 mL/min; Visit 3: 416.69 ± 49.90 mL/min; F(2, 38) = 1.29, p = 0.287, η²_p_ = 0.06), indicating comparable pre-trial resting metabolic status across periods.

### 4.1 Exercise-phase substrate oxidation and energy expenditure

**Figure 2:**
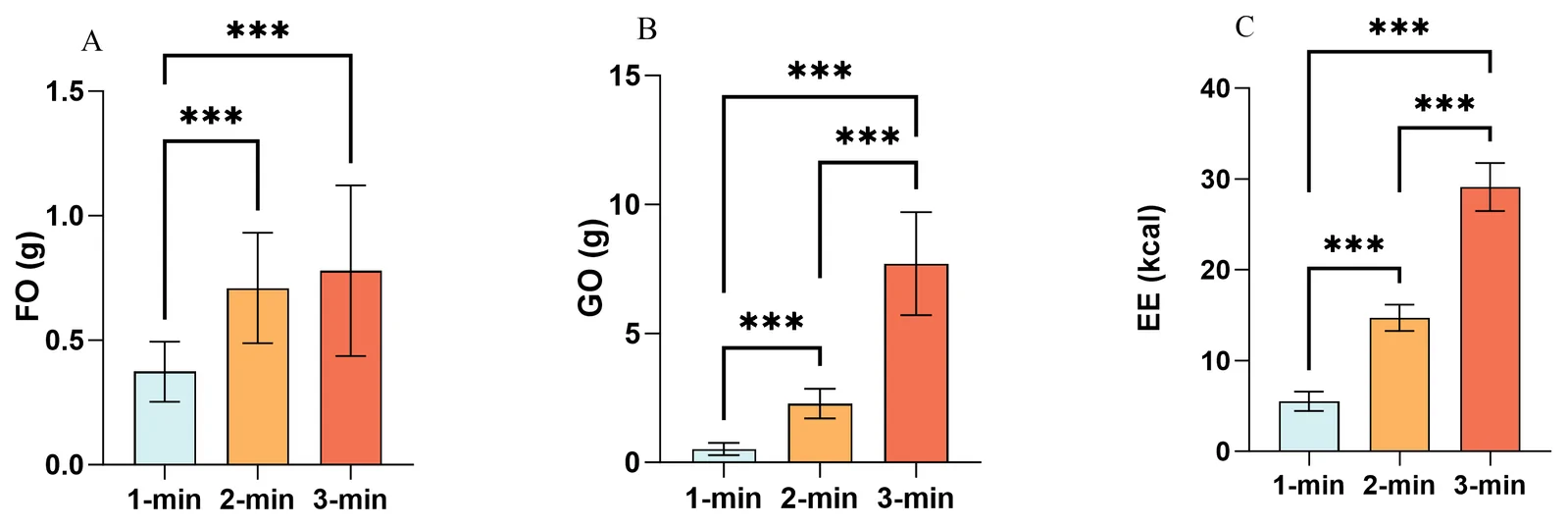
**(A)** fat oxidation (FO), **(B)** glucose oxidation (GO), and **(C)**energy expenditure (EE) during the exercise period following the 1-min, 2-min, and 3-min exercise snack protocols. Data are presented as mean ± SD (n = 20). ***p < 0.001.

Fat oxidation differed significantly among the three protocols (F(1.43, 27.11) = 34.34, p < 0.001, η²_p_ = 0.64). Fat oxidation increased with exercise duration. Bonferroni *post hoc* analyses showed that fat oxidation was significantly higher in the 2-min and 3-min protocols than in the 1-min protocol (both p < 0.001), whereas no significant difference was observed between the 2-min and 3-min protocols (**Figure 2A**).

Glucose oxidation differed significantly among the three protocols (F(1.14, 21.59) = 230.00, p < 0.001, η²_p_ = 0.92). Glucose oxidation increased with exercise duration, with significant differences across all pairwise comparisons (p < 0.001) (**Figure 2B**).

EE differed significantly among the three protocols (F(1.31, 24.79) = 1001.35, p < 0.001, η²_p_ = 0.98). EE increased with exercise duration, with significant differences across all pairwise comparisons (p < 0.001) (**Figure 2C**; **Table 1**).

**Table 1:** Cumulative fat oxidation (FO), glucose oxidation (GO), and energy expenditure (EE) during the exercise and recovery periods following the 1-min, 2-min, and 3-min exercise snack (ES) protocols.

Exercise period	1-min ES	2-min ES	3-min ES	95% CI
FO (g)	0.37 ± 0.12	0.71 ± 0.22	0.78 ± 0.34	1-min: [0.32, 0.43] 2-min: [0.61, 0.81] 3-min: [0.62, 0.94]
GO (g)	0.52 ± 0.24	2.28 ± 0.58	7.71 ± 2.00	1-min: [0.41, 0.63] 2-min: [2.01, 2.55] 3-min: [6.77, 8.64]
EE (kcal)	5.51 ± 1.07	14.70 ± 1.45	29.12 ± 2.65	1-min: [5.01, 6.01] 2-min: [14.02, 15.38] 3-min: [27.88, 30.36]
10-minute recovery period				
FO (g)	0.73 ± 0.45	0.23 ± 0.15	0.20 ± 0.10	1-min: [0.52, 0.94] 2-min: [0.16, 0.30] 3-min: [0.15, 0.24]
GO (g)	7.05 ± 1.95	15.49 ± 2.17	19.91 ± 3.03	1-min: [6.13, 7.96] 2-min: [14.48, 16.51] 3-min: [18.49, 21.33]
EE (kcal)	27.75 ± 2.76	35.21 ± 3.96	41.11 ± 3.52	1-min: [26.46, 29.05] 2-min: [33.36, 37.07] 3-min: [39.46, 42.76]
20-minute recovery period				
FO (g)	1.46 ± 0.70	1.25 ± 0.51	1.27 ± 0.53	1-min: [1.13, 1.79] 2-min: [1.01, 1.49] 3-min: [1.02, 1.52]
GO (g)	10.64 ± 2.87	18.97 ± 2.74	24.01 ± 3.85	1-min: [9.29, 11.98] 2-min: [17.69, 20.26] 3-min: [22.21, 25.81]
EE (kcal)	48.51 ± 6.07	56.91 ± 6.56	64.74 ± 5.89	1-min: [45.67, 51.35] 2-min: [53.84, 59.99] 3-min: [61.98, 67.49]
30-minute recovery period				
FO (g)	2.49 ± 0.94	2.48 ± 0.74	2.70 ± 0.84	1-min: [2.05, 2.93] 2-min: [2.13, 2.83] 3-min: [2.31, 3.10]
GO (g)	13.21 ± 2.96	21.41 ± 3.05	26.85 ± 4.32	1-min: [11.83, 14.60] 2-min: [19.99, 22.84] 3-min: [24.83, 28.87]
EE (kcal)	65.10 ± 7.13	76.07 ± 9.08	86.08 ± 8.12	1-min: [61.77, 68.44] 2-min: [71.82, 80.32] 3-min: [82.28, 89.89]

Data are presented as mean ± SD (n = 20). The 95% CIs are based on estimated marginal means from the repeated-measures ANOVA.

### 4.2 Post-exercise recovery phase

To further evaluate the persistence of post-exercise metabolic activation, changes in EER were analyzed throughout the 30-min recovery period.

#### 4.2.1 Post-exercise recovery energy expenditure rate

EER during post-exercise recovery showed a significant main effect of exercise protocol (F(2, 38) = 27.68, p < 0.001, η²_p_ = 0.59), a significant main effect of time (F(5, 95) = 121.23, p < 0.001, η²_p_ = 0.87), and a significant protocol × time interaction (F(10, 190) = 7.12, p < 0.001, η²_p_ = 0.27), indicating that the temporal pattern of EER during recovery differed significantly among the three protocols. Mean recovery EER was significantly higher in the 3-min protocol than in the 1-min and 2-min protocols (both p < 0.001), and was significantly higher in the 2-min protocol than in the 1-min protocol (p = 0.008).

**Figure 3:**
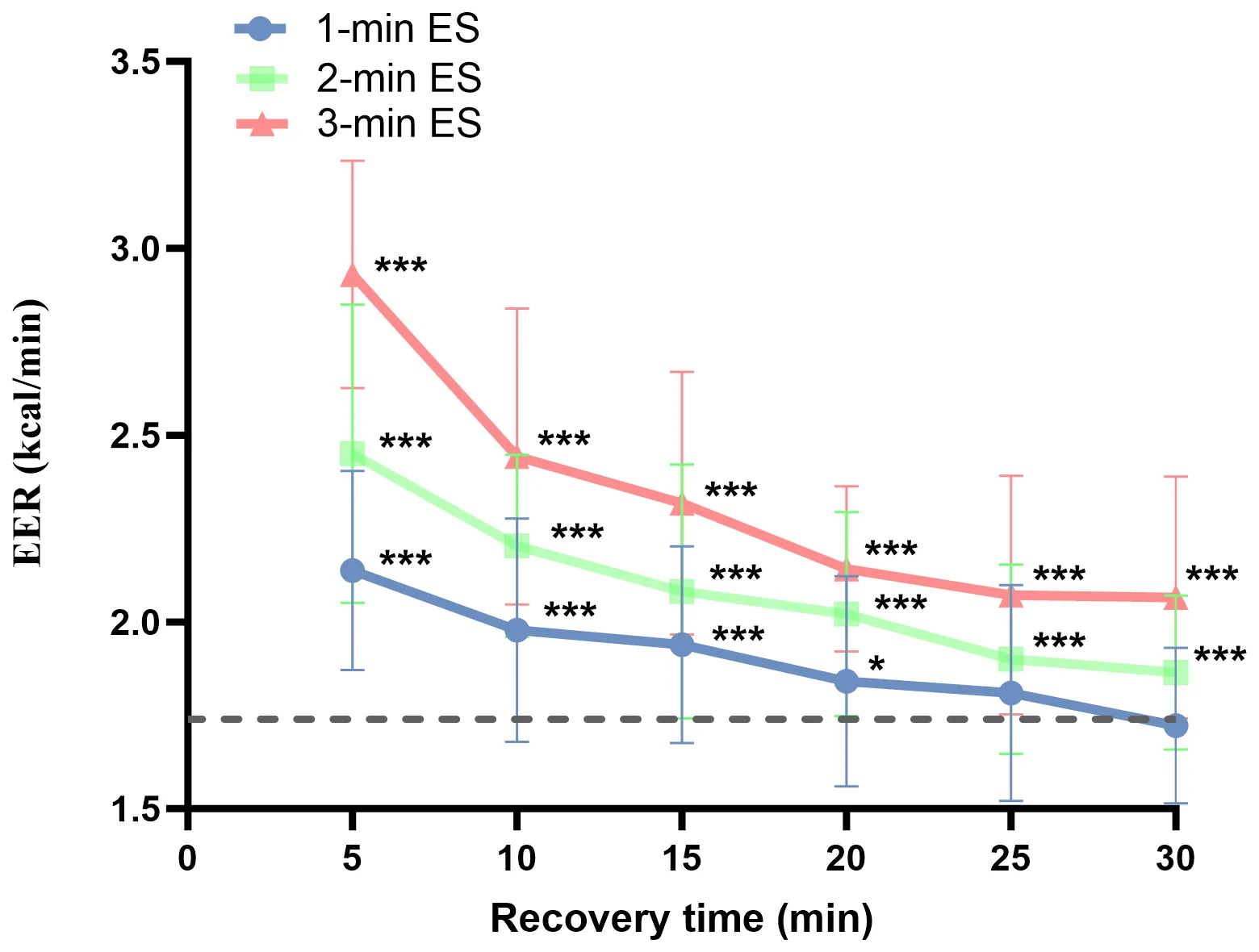
Changes in energy expenditure rate (EER) at 5, 10, 15, 20, 25, and 30 min during the recovery period following the 1-min, 2-min, and 3-min exercise snack (ES) protocols. Data are presented as mean ± SD (n = 20). The horizontal dashed line represents the mean resting EER averaged across the three exercise snack protocols. Asterisks indicate EER values significantly higher than the corresponding resting value within the same ES protocol (*p < 0.05, ***p < 0.001).

EER gradually decreased over the course of recovery in all three protocols. In the 1-min protocol, EER was no longer significantly different from the resting level after 25 min of recovery (p = 0.095). In contrast, EER remained significantly higher than the resting level throughout the 30-min recovery period in both the 2-min and 3-min protocols (both p < 0.001) (**Figure 3**).

#### 4.2.2 Post-exercise substrate oxidation rate

**Figure 4:**
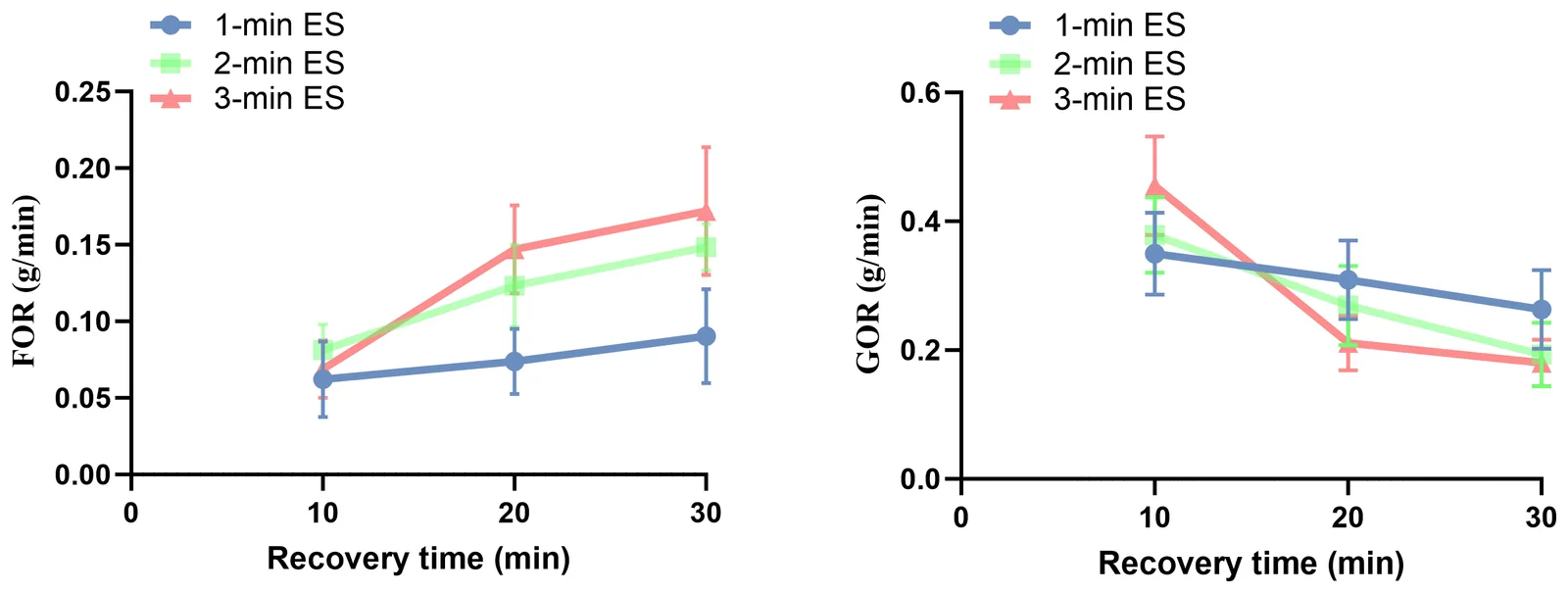
Changes in fat oxidation and glucose oxidation rates at 10, 20, and 30 min during the recovery period following 1-min, 2-min, and 3-min exercise snack (ES) protocols. Data are presented as mean ± SD (n = 20). FOR, fat oxidation rate; GOR, glucose oxidation rate.

FOR during post-exercise recovery showed a significant main effect of exercise protocol (F(2, 38) = 68.40, p < 0.001, η²_p_ = 0.78), a significant main effect of time (F(2, 38) = 156.80, p < 0.001, η²_p_ = 0.89), and a significant protocol × time interaction (F(4, 76) = 24.86, p < 0.001, η²_p_ = 0.57), with the temporal pattern of FOR differing among protocols. FOR increased progressively over the course of recovery in all three protocols, although the magnitude of the increase differed among protocols. At 30 min of recovery, FOR was significantly higher in the 2-min and 3-min protocols than in the 1-min protocol (both p < 0.001), whereas no significant difference was observed between the 2-min and 3-min protocols (p = 0.068) (**Figure 4**).

GOR during post-exercise recovery showed no significant main effect of exercise protocol (F(2, 38) = 1.90, p = 0.163, η²_p_ = 0.09), but a significant main effect of time (F(2, 38) = 234.15, p < 0.001, η²_p_ = 0.92) and a significant protocol × time interaction (F(4, 76) = 38.42, p < 0.001, η²_p_ = 0.67), indicating that the temporal pattern of GOR during recovery differed among the three protocols. GOR gradually decreased over the course of recovery in all three protocols (**Figure 4**; **Table 2**).

**Table 2:** Rate of substrate oxidation and energy expenditure at 10, 20, and 30 min after exercise.

Variable	1-min ES	2-min ES	3-min ES
Recovery 10 min			
FOR (g/min)	0.06 ± 0.02	0.08 ± 0.02	0.07 ± 0.02
GOR (g/min)	0.35 ± 0.06	0.38 ± 0.06	0.46 ± 0.08
EER (kcal/min)	1.98 ± 0.30	2.20 ± 0.24	2.44 ± 0.40
Recovery 20 min			
FOR (g/min)	0.07 ± 0.02	0.12 ± 0.02	0.14 ± 0.03
GOR (g/min)	0.31 ± 0.06	0.27 ± 0.06	0.21 ± 0.04
EER (kcal/min)	1.84 ± 0.28	2.02 ± 0.27	2.14 ± 0.22
Recovery 30 min			
FOR (g/min)	0.09 ± 0.03	0.15 ± 0.02	0.17 ± 0.04
GOR (g/min)	0.27 ± 0.06	0.21 ± 0.07	0.18 ± 0.04
EER (kcal/min)	1.72 ± 0.21	1.87 ± 0.21	2.07 ± 0.32

FOR, fat oxidation rate; GOR, glucose oxidation rate; EER, energy expenditure rate; ES, exercise snack. Data are presented as mean ± SD (n = 20).

#### 4.2.3 Recovery-period substrate oxidation and EE

To further compare cumulative substrate utilization and EE during recovery among the three protocols, cumulative fat oxidation, glucose oxidation, and EE were calculated at 10, 20, and 30 min of recovery.

##### 4.2.3.1 10-min recovery

**Figure 5:**
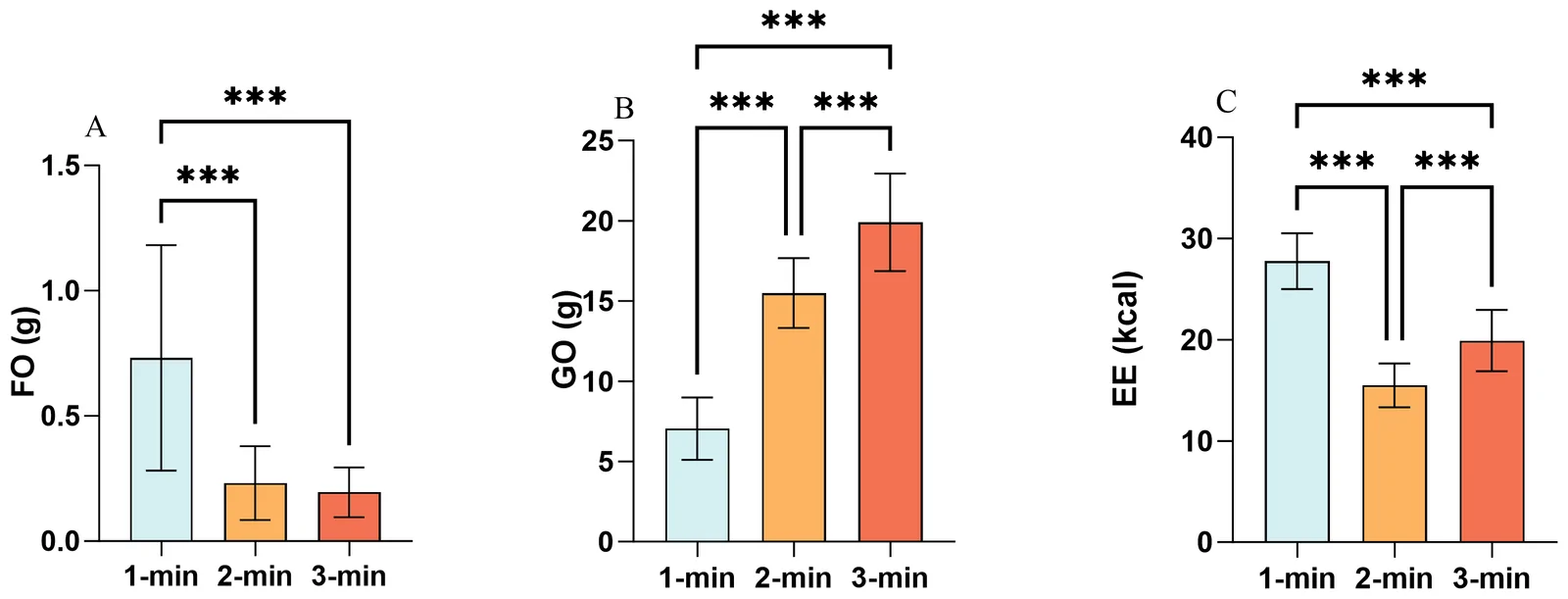
**(A)** fat oxidation (FO), **(B)** glucose oxidation (GO), and **(C)** energy expenditure (EE) during the 10-min recovery period following the 1-min, 2-min, and 3-min exercise snack protocols. Data are presented as mean ± SD (n = 20). ***p < 0.001.

Fat oxidation differed significantly among the three protocols (F(1.17, 22.14) = 29.45, p < 0.001, η²_p_ = 0.61). Fat oxidation decreased with increasing exercise duration, with the 1-min protocol showing the highest fat oxidation. Bonferroni *post hoc* analyses revealed significant differences between the 1-min and 2-min protocols and between the 1-min and 3-min protocols (both p < 0.001), whereas no significant difference was observed between the 2-min and 3-min protocols (**Figure 5A**). Glucose oxidation differed significantly among the three protocols (F(2, 38) = 258.98, p < 0.001, η²_p_ = 0.93). Glucose oxidation increased with exercise duration, with significant differences across all pairwise comparisons (p < 0.001) (**Figure 5B**). EE differed significantly among the three protocols (F(2, 38) = 145.64, p < 0.001, η²_p_ = 0.89). The 3-min protocol showed the highest EE. All pairwise comparisons were significant (p < 0.001) (**Figure 5C**).

##### 4.2.3.2 20-min recovery

**Figure 6:**
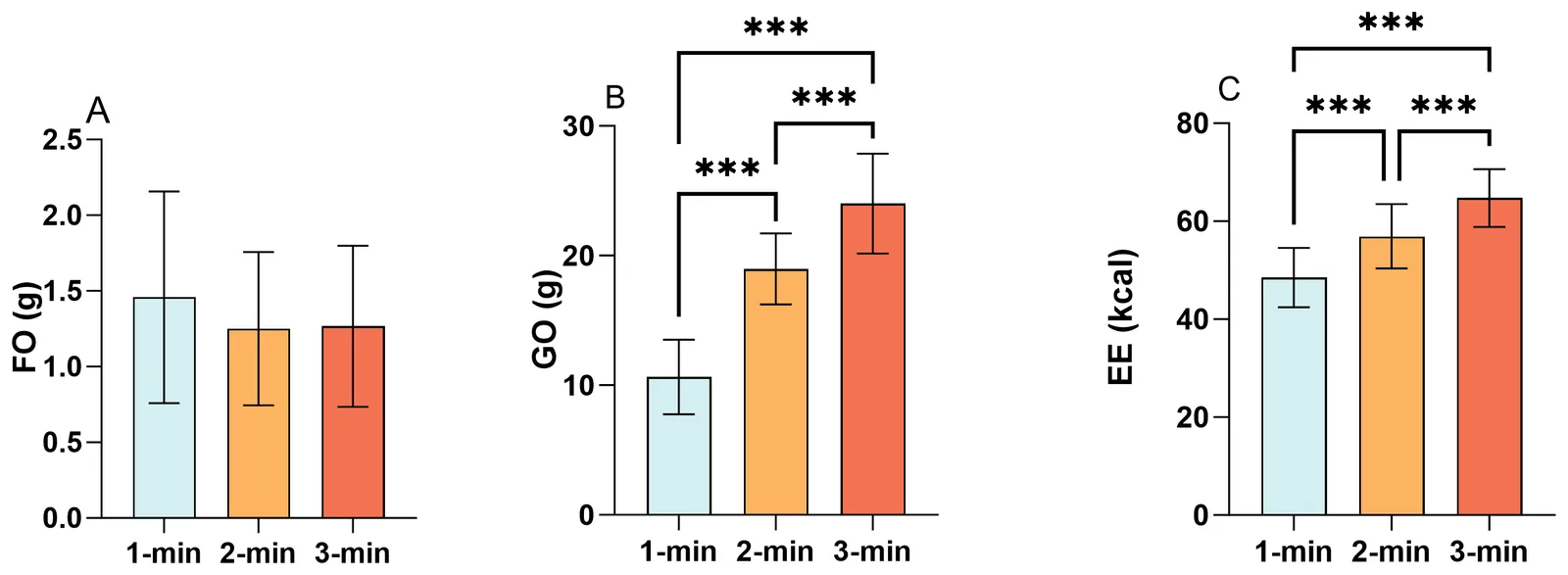
**(A)** fat oxidation (FO), **(B)** glucose oxidation (GO), and **(C)** energy expenditure (EE) during the 20-min recovery period following the 1-min, 2-min, and 3-min exercise snack protocols. Data are presented as mean ± SD (n = 20). ***p < 0.001.

Fat oxidation did not differ significantly among the three protocols (F(2, 38) = 1.32, p > 0.05, η²_p_ = 0.07) (**Figure 6A**). Glucose oxidation differed significantly among the three protocols (F(2, 38) = 126.24, p < 0.001, η²_p_ = 0.87). Glucose oxidation increased with exercise duration, with significant differences across all pairwise comparisons (all p < 0.001) (**Figure 6B**). EE differed significantly among the three protocols (F(1.53, 29.05) = 62.40, p < 0.001, η²_p_ = 0.77). EE increased with exercise duration, with significant differences across all pairwise comparisons (all p < 0.001) (**Figure 6C**).

##### 4.2.3.3 30-min recovery

**Figure 7:**
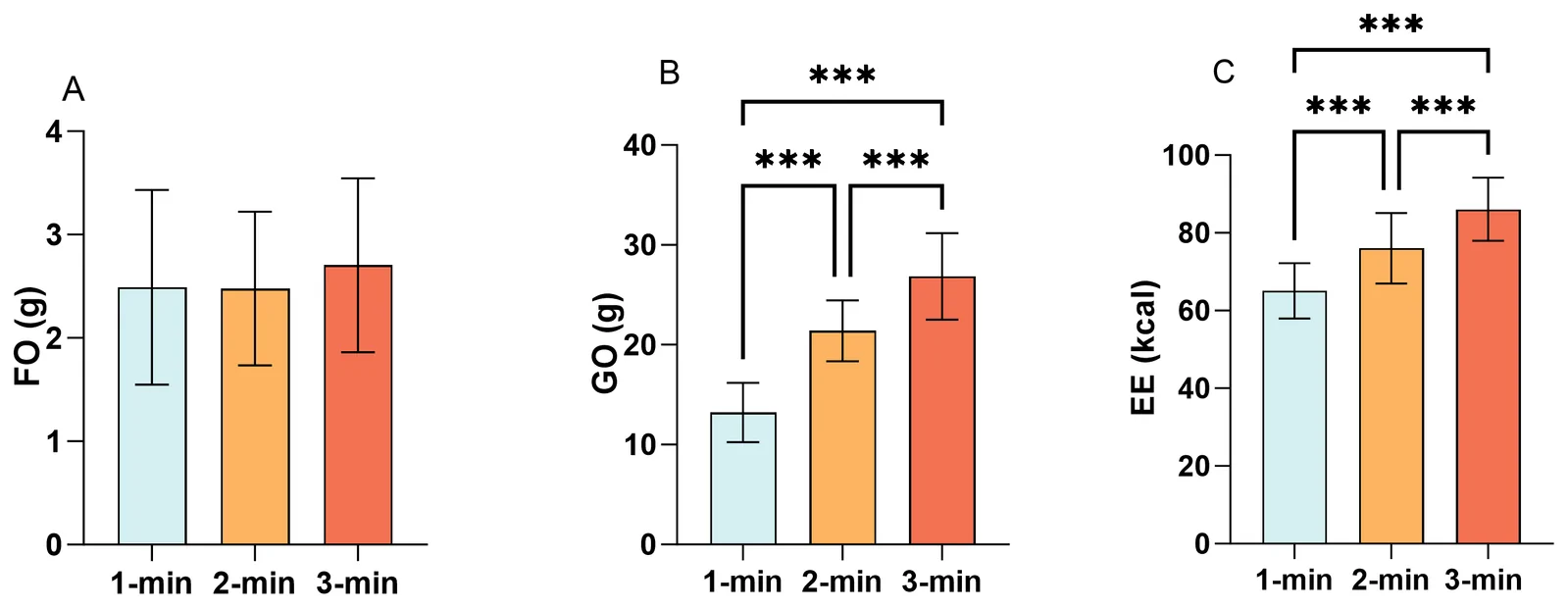
**(A)** fat oxidation (FO), **(B)** glucose oxidation (GO), and **(C)** energy expenditure (EE) during the 30-min recovery period following the 1-min, 2-min, and 3-min exercise snack protocols. Data are presented as mean ± SD (n = 20). ***p < 0.001.

Fat oxidation did not differ significantly among the three protocols (F(2, 38) = 0.75, p > 0.05, η²_p_ = 0.04) (**Figure 7A**). Glucose oxidation differed significantly among the three protocols (F(2, 38) = 106.74, p < 0.001, η²_p_ = 0.85). Glucose oxidation increased with exercise duration, with significant differences across all pairwise comparisons (p < 0.001) (**Figure 7B**). EE differed significantly among the three protocols (F(1.50, 28.41) = 128.75, p < 0.001, η²_p_ = 0.82). EE increased with exercise duration, with significant differences across all pairwise comparisons (all p < 0.001) (**Figure 7C**; **Table 1**).

### 4.3 Heart rate

HR differed significantly among the three protocols during exercise (F(2, 38) = 114.83, p < 0.001, η²_p_ = 0.86) and at 10 min (F(1.48, 28.17) = 62.91, p < 0.001, η²_p_ = 0.77), 20 min (F(1.35, 25.64) = 44.81, p < 0.001, η²_p_ = 0.70), and 30 min (F(1.40, 26.65) = 35.35, p < 0.001, η²_p_ = 0.65) of recovery. Pairwise comparisons were significant among all three protocols at each time point (p < 0.001 during exercise and at 10 and 20 min; p < 0.01 at 30 min) (**Table 3**).

**Table 3:** Resting, exercise, and post-exercise heart rates following the 1-min, 2-min, and 3-min exercise snack (ES) protocols.

Variable	Resting HR	During exercise HR	HRT_10_	HRT_20_	HRT_30_
1-min ES	85.11 ± 14.63	109.75 ± 12.10	92.59 ± 16.08	89.65 ± 14.66	87.33 ± 13.70
2-min ES	82.71 ± 12.18	124.21 ± 9.44	104.50 ± 16.18	98.82 ± 14.57	95.07 ± 13.66
3-min ES	79.84 ± 12.15	139.25 ± 12.89	117.67 ± 18.07	109.66 ± 16.66	104.07 ± 15.27
p value	p = 0.054	p < 0.001	p < 0.001	p < 0.001	p < 0.001

Data are presented as mean ± SD (n = 20).

HRT_10_, heart rate during the 10-min recovery period; HRT_20_, heart rate during the 20-min recovery period; HRT_30_, heart rate during the 30-min recovery period.

### 4.4 Ratings of perceived exertion

RPE differed significantly among the three protocols during exercise (F(2, 38) = 91.80, p < 0.001, η²_p_ = 0.83) and at 10 min (F(2, 38) = 48.09, p < 0.001, η²_p_ = 0.72), 20 min (F(2, 38) = 47.12, p < 0.001, η²p = 0.71), and 30 min (F(2, 38) = 45.07, p < 0.001, η²_p_ = 0.70) of recovery. Bonferroni *post hoc* analyses showed significant differences across all pairwise comparisons during exercise and at each recovery time point (all p < 0.001) (**Table 4**).

**Table 4:** Ratings of perceived exertion during exercise and recovery periods following the 1-min, 2-min, and 3-min exercise snack (ES) protocols.

Variable	During exercise	R10	R20	R30
1-min ES	11.05 ± 1.10	9.35 ± 1.76	8.95 ± 1.50	8.70 ± 1.30
2-min ES	12.40 ± 1.10	10.40 ± 1.64	9.85 ± 1.50	9.60 ± 1.27
3-min ES	13.85 ± 1.31	11.70 ± 1.87	11.15 ± 1.42	10.55 ± 1.23
p value	p < 0.001	p < 0.001	p < 0.001	p < 0.001

R10, 10-min recovery period; R20, 20-min recovery period; R30, 30-min recovery period. Data are presented as mean ± SD (n = 20).

### 4.5 Respiratory exchange ratio

During exercise, respiratory exchange ratio (RER) differed significantly among the three protocols (F(2, 38) = 70.27, p < 0.001, η²_p_ = 0.79). All pairwise comparisons were significant (all p < 0.01).

**Figure 8:**
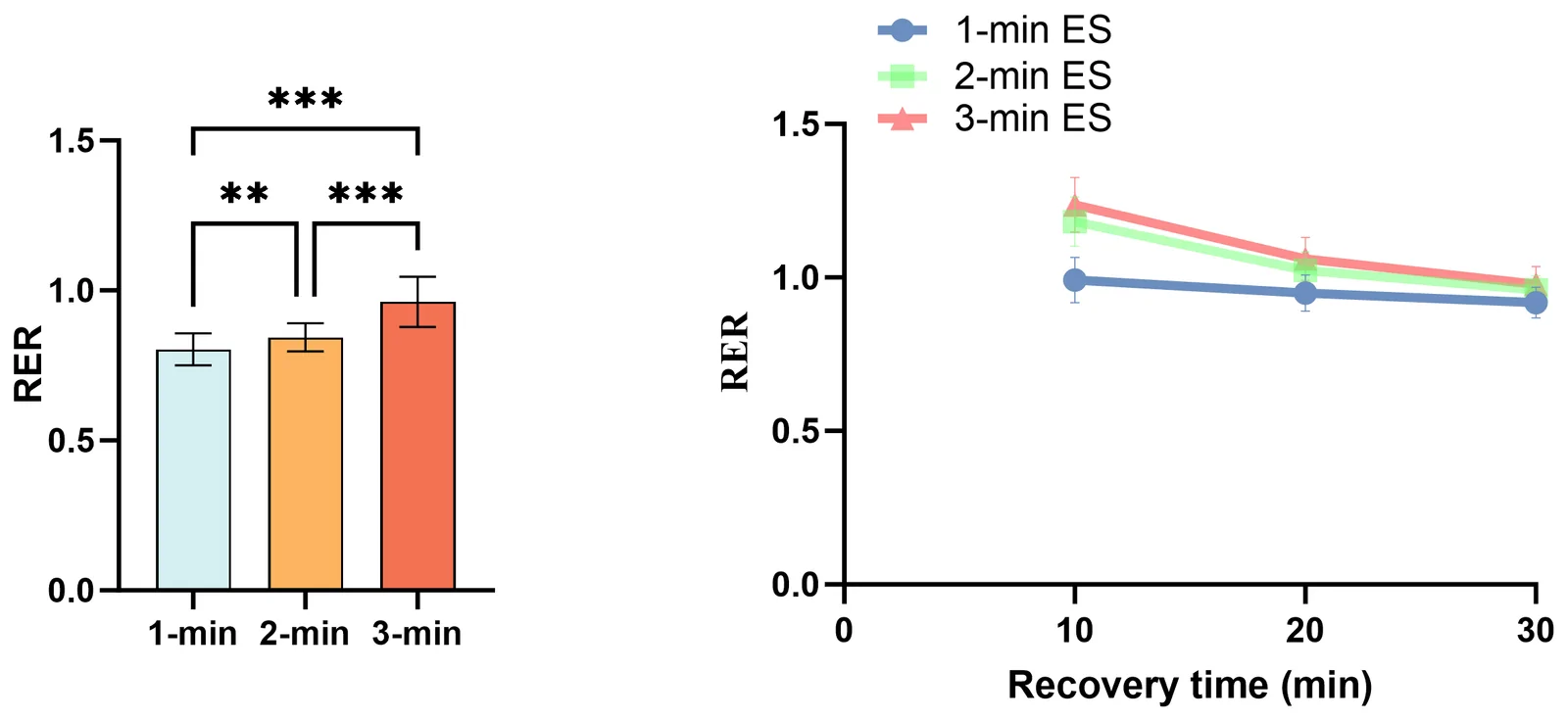
Respiratory exchange ratio (RER) during exercise and the 30-min recovery period following the 1-min, 2-min, and 3-min exercise snack (ES) protocols. The left panel shows mean RER during exercise, and the right panel shows RER at 10, 20, and 30 min during recovery. Data are presented as mean ± SD (n = 20). **p < 0.01, ***p < 0.001.

During post-exercise recovery, RER showed a significant main effect of exercise protocol (F(2, 38) = 68.97, p < 0.001, η²_p_ = 0.78), a significant main effect of time (F(2, 38) = 454.05, p < 0.001, η²_p_ = 0.96), and a significant protocol × time interaction (F(4, 76) = 170.39, p < 0.001, η²_p_ = 0.90). Mean RER during recovery differed significantly between the 1-min and 2-min protocols and between the 1-min and 3-min protocols (both p < 0.001), whereas no significant difference was observed between the 2-min and 3-min protocols (p = 0.054). RER gradually decreased over the course of recovery in all three protocols, although the magnitude of the decrease differed among protocols (**Figure 8**; **Table 5**).

**Table 5:** Respiratory exchange ratio (RER) during exercise and recovery following the 1-min, 2-min, and 3-min exercise snack (ES) protocols.

Variable	During exercise	RER_10_	RER_20_	RER_30_
1-min ES	0.80 ± 0.05	0.99 ± 0.07	0.95 ± 0.06	0.92 ± 0.05
2-min ES	0.84 ± 0.05	1.18 ± 0.08	1.02 ± 0.06	0.96 ± 0.05
3-min ES	0.96 ± 0.08	1.24 ± 0.09	1.06 ± 0.07	0.98 ± 0.06
p value	p < 0.001	p < 0.001	p < 0.001	p < 0.001

RER_10_, respiratory exchange ratio during the 10-min recovery period; RER_20_, respiratory exchange ratio during the 20-min recovery period; RER_30_, respiratory exchange ratio during the 30-min recovery period. Data are presented as mean ± SD (n = 20).

## 5 Discussion

Previous studies have demonstrated that exercise snacks can elicit acute physiological and metabolic responses; however, direct evidence comparing the effects of different exercise durations on EE and substrate oxidation during exercise and post-exercise recovery remains limited. Under standardized incremental loading and peak power output conditions, the present study systematically compared EE and substrate metabolism in response to 1-, 2-, and 3-min exercise snack protocols. The results demonstrated that all short-duration exercise snack protocols elicited significant changes in EE and substrate oxidation within a relatively short period, and that the overall magnitude of these changes differed among the exercise-duration protocols and was generally greater in the longer-duration protocols. However, the responses of individual metabolic variables were not entirely consistent across protocols, as several variables did not show statistically significant differences between the 2-min and 3-min protocols. These findings further expand the current evidence regarding the metabolic characteristics of short-duration exercise snacks and provide experimental support for optimizing time-efficient exercise interventions for sedentary adults.

### 5.1 Substrate metabolism and energy expenditure characteristics during exercise

The present findings demonstrate that substrate utilization differed significantly among the three exercise-duration protocols, with distinct patterns observed in fat oxidation, glucose oxidation, and EE. During exercise, fat oxidation was greater in the 2-min and 3-min protocols than in the 1-min protocol, while the fat oxidation response following the 2-min protocol was not significantly different from that observed following the 3-min protocol. This pattern is consistent with the multifactorial regulation of fat oxidation by exercise intensity, duration, and fatty acid availability and uptake (Roepstorff et al., 2004).

Glucose oxidation and total EE increased with exercise duration, and RER was higher in the 3-min than in the 2-min protocol. Together, these findings indicate a greater relative contribution of carbohydrate oxidation to energy provision as exercise demand increased. Physiologically, greater energy demand favors rapid ATP provision through carbohydrate metabolism, whereas fat oxidation does not necessarily increase proportionally. Thus, the extra EE accumulated during the additional 1 min of exercise in the 3-min protocol may have been supported predominantly by greater carbohydrate oxidation rather than by a proportional increase in fat oxidation. This interpretation is consistent with previous evidence showing that carbohydrate utilization increases with increasing exercise intensity, whereas whole-body fat oxidation may plateau or decline at higher intensities (Romijn et al., 1993; van Loon et al., 2001). Accordingly, the fat oxidation response following the 2-min protocol was not significantly different from that observed following the 3-min protocol, whereas the additional minute of exercise was associated primarily with greater carbohydrate utilization and total EE. Taken together, the large effect sizes observed for fat oxidation, glucose oxidation, and EE further indicate substantial differences in the magnitude of the acute metabolic responses among the three exercise-duration protocols.

From the perspective of physiological load, HR and RPE increased with exercise duration, indicating progressively greater cardiovascular and perceptual demands. The progressive elevation in HR may be attributed to the activation of the exercise pressor reflex mediated by mechanoreceptors and enhanced sympathetic nervous system activity during exercise (Michael et al., 2017), reflecting the increased demand for oxygen delivery and circulatory regulation associated with prolonged exercise duration and greater cumulative workload (Tanoue et al., 2022; Hori et al., 2023). In addition, the changes in RPE closely paralleled those in HR and EE, indicating that perceived exertion reflected, to some extent, changes in physiological load and metabolic demand during short-duration exercise snacks.

Although the loading protocol progressed to a target workload of 180 W, the overall cardiovascular and perceptual responses observed during the formal trials did not indicate maximal exertion in the present participants. Mean exercise HR ranged from 109.75 to 139.25 beats/min and mean RPE ranged from 11.05 to 13.85 across the three exercise conditions, indicating physiological responses consistent with a tolerable submaximal load at the group level. In addition, the standardized protocol elicited measurable changes in EE across the exercise-duration conditions, supporting its adequacy for inducing detectable acute metabolic responses. Nevertheless, because HR represented the mean response across the entire exercise bout rather than the response specifically at 180 W, these findings should be interpreted as evidence of the overall tolerability of the protocol rather than as a direct classification of the relative intensity of 180 W. The common absolute target workload was selected because the primary aim of the present study was to compare EE and substrate utilization among the 1-, 2-, and 3-min exercise-duration protocols under the same standardized loading progression. Maintaining a consistent external exercise stimulus facilitated direct within-subject comparisons among the 1-, 2-, and 3-min conditions. Individual differences in body size and fitness level should nevertheless be considered when interpreting the relative physiological demands imposed by the same absolute workload. It should also be noted that, under this design, longer exercise duration was accompanied by greater total work performed and a longer time spent at the target workload; therefore, the observed differences should be interpreted as responses to the respective exercise-duration protocols rather than as the isolated effect of exercise duration alone.

Exercise snacks have been recognized as a time-efficient exercise strategy that may improve cardiorespiratory fitness in sedentary or physically inactive populations, with recent studies also supporting their feasibility (Yin et al., 2024b; Wang T. et al. (2024); Weston et al., 2025; Yin et al., 2024a). Complementing this broader evidence, recent acute research has shown that even 1–3 min of stair climbing and descending can reduce postprandial glucose and insulin in young adults, with 3 min also improving insulin sensitivity (Moore et al., 2024). The present study adds to this literature by directly comparing 1-, 2-, and 3-min cycling exercise snacks under the same standardized incremental loading protocol and by examining acute changes in EE and substrate utilization during both exercise and recovery. Within the present sample, these findings suggest that a 2-min exercise snack may represent a time-efficient strategy for eliciting acute changes in EE and substrate utilization.

### 5.2 Substrate metabolism and energy expenditure characteristics during recovery

Recovery metabolism is an important component in evaluating the post-exercise metabolic effects of short-duration exercise snacks. Previous studies have demonstrated that post-exercise fat oxidation, EE, and cardiovascular responses during recovery are closely associated with exercise intensity and duration (Warren et al., 2009). Under standardized incremental loading and peak power output-controlled conditions, the present study further revealed differences among the exercise-duration protocols in recovery EE, the duration of post-exercise metabolic activation, and substrate oxidation profiles. The large protocol × time effect sizes observed for the recovery metabolic variables further indicate meaningful differences among the protocols in the temporal pattern of post-exercise metabolic recovery.

Recovery EER declined across all conditions but remained elevated for longer following the 2- and 3-min protocols than following the 1-min protocol, indicating greater persistence of post-exercise energy demand in the longer-duration protocols. Previous studies have suggested that EPOC is a major physiological mechanism underlying elevated post-exercise EE, and that both its magnitude and duration are influenced by exercise intensity, duration, and exercise modality. EPOC may involve ATP-PCr resynthesis, lactate clearance, thermoregulation, sustained sympathetic nervous system activity, and glycogen resynthesis (Børsheim and Bahr, 2003; Panissa et al., 2021; Katz, 2022). These EPOC-related processes provide a plausible physiological explanation for the more prolonged elevation in recovery EE following the longer exercise protocols, reflecting continued energy requirements associated with the restoration of physiological homeostasis.

During recovery, FOR progressively increased, whereas GOR declined, indicating a time-dependent change in the pattern of substrate oxidation. At 30 min of recovery, FOR remained higher in the 2-min and 3-min protocols than in the 1-min protocol, with no significant difference between the 2-min and 3-min protocols. Interestingly, cumulative fat oxidation during the first 10 min of recovery was greater following the 1-min protocol than following the 2- and 3-min protocols; however, this difference was no longer significant at 20 or 30 min. This transient pattern may reflect differences in early post-exercise substrate utilization, as the longer protocols elicited greater carbohydrate oxidation and higher RER during early recovery, which may indicate a relatively delayed transition toward greater lipid utilization. Previous studies have demonstrated that the increase in post-exercise fat oxidation is typically accompanied by a redistribution of substrate utilization during recovery, which may be associated with enhanced catecholamine-mediated fatty acid mobilization and post-exercise glycogen repletion (Long et al., 2008; Lundsgaard et al., 2020). This finding is consistent with the “crossover concept” proposed by Brooks and Mercier, which suggests that the relative contributions of carbohydrate and fat to energy production are dynamically regulated according to the body’s metabolic state (Brooks and Mercier, 1994). Previous studies have suggested that this pattern represents an important metabolic characteristic of the post-exercise recovery period and is closely related to the restoration of substrate and energy homeostasis (Lundsgaard et al., 2020).

Although HR remained above baseline throughout the 30-min recovery period following the 2- and 3-min protocols, HR progressively declined during recovery in all three exercise conditions. Post-exercise HR recovery reflects the gradual restoration of cardiovascular autonomic regulation after exercise, and its time course is influenced by the magnitude of the preceding exercise stimulus (Peçanha et al., 2017; Facioli et al., 2021). Accordingly, the residual elevation in HR at 30 min is more consistent with an ongoing physiological recovery process than with a sustained or progressively increasing cardiovascular response. In addition, the overall exercise HR and RPE responses did not suggest near-maximal or maximal exertion at the group level. Taken together, these observations support the short-term physiological tolerability of the protocol in the sedentary young men included in this study.

Consistent with these cardiovascular recovery patterns, RPE and RER also showed distinct recovery responses following exercise snacks of different durations. Longer exercise durations were associated with higher RPE during recovery, suggesting greater residual perceived exertion. Meanwhile, RER progressively declined during recovery, consistent with the increase in FOR and the decrease in GOR, further supporting time-dependent changes in substrate oxidation during recovery. These findings suggest that the practical application of short-duration exercise snacks should consider not only EE and substrate utilization but also subjective fatigue, exercise tolerance, and long-term adherence (Birnbaumer et al., 2022).

Taken together, the exercise and recovery findings indicate distinct time-dependent patterns of substrate oxidation, with greater carbohydrate oxidation as exercise demand increased and progressively increasing fat oxidation during recovery. Thus, the physiological effects of short-duration exercise snacks reflect not only the immediate energy cost of exercise but also the persistence of post-exercise energy demand and the temporal evolution of substrate oxidation during recovery. From a practical perspective, fat oxidation did not differ significantly between the 2-min and 3-min protocols, while the 2-min protocol required less exercise time, and both protocols maintained elevated EE during recovery. These findings suggest that a 2-min exercise snack may provide a time-efficient approach for eliciting meaningful acute metabolic responses in sedentary individuals.

### 5.3 Study limitations and future directions

This study included only young sedentary male college students and did not include females, individuals from other age groups, or those with different fitness levels. Accordingly, the applicability of these findings across sexes remains to be established. The restricted participant characteristics, together with the relatively small sample size, may limit the generalizability of the findings. Sedentary status was determined based on self-reported habitual behavior during recruitment rather than objective quantification of sedentary time, which may limit the precision of participant characterization. Because exercise duration, total work performed, and time spent at the target workload varied concurrently across the three protocols, the observed differences should be interpreted as differences among the exercise-duration protocols rather than as effects attributable exclusively to exercise duration. In addition, recovery was monitored only for 30 min after exercise, and metabolic responses beyond the 30-min recovery period were not evaluated. Biochemical metabolic markers, such as blood glucose, insulin, lactate, and hormonal responses, were not assessed, which limits further interpretation of the underlying metabolic mechanisms. Although Bonferroni adjustment was applied to *post hoc* pairwise comparisons to reduce the risk of Type I error, the relatively large number of statistical comparisons across multiple outcomes and recovery assessments should still be considered when interpreting individual significant findings. Furthermore, this study employed an acute single-session intervention and therefore did not examine the effects of long-term repeated exercise snack interventions on metabolic adaptations or changes in body composition. Future studies should recruit female participants, individuals across different age groups, and those with diverse fitness levels. In addition, long-term intervention designs incorporating assessments of body composition, blood glucose, blood lipids, and insulin sensitivity are warranted to further clarify the long-term effects of short-duration exercise snacks on metabolic health and the underlying mechanisms.

## 6 Conclusion

The present study demonstrates that short-duration exercise snacks of different durations elicit marked acute energy metabolic and substrate oxidation responses in sedentary male college students. During exercise, the 2-min protocol significantly increased fat oxidation, with no significant difference in fat oxidation compared with the 3-min protocol. During recovery, both the 2-min and 3-min protocols maintained EER at levels significantly above resting values throughout the entire 30-min recovery period, with no significant difference in fat oxidation between the two protocols at 30 min of recovery. These findings suggest that a 2-min exercise snack may be sufficient to enhance energy metabolism and fat oxidation while eliciting sustained post-exercise metabolic activation. These findings provide experimental evidence supporting the potential application of exercise snacks in sedentary adults and the optimization of time-efficient exercise intervention strategies.

## Data Availability

The raw data supporting the conclusions of this article will be made available by the authors, without undue reservation.
